# Exercise Rescues Gene Pathways Involved in Vascular Expansion and Promotes Functional Angiogenesis in Subcutaneous White Adipose Tissue

**DOI:** 10.3390/ijms20082046

**Published:** 2019-04-25

**Authors:** So Yun Min, Heather Learnard, Shashi Kant, Olga Gealikman, Raziel Rojas-Rodriguez, Tiffany DeSouza, Anand Desai, John F. Keaney, Silvia Corvera, Siobhan M. Craige

**Affiliations:** 1 Program in Molecular Medicine, University of Massachusetts Medical School, Worcester, MA 01655, USA; ssso1019@catholic.ac.kr (S.Y.M.); Olgagealik@gmail.com (O.G.); Raziel.Rojas-Rodriguez@umassmed.edu (R.R.-R.); Tiffany.DeSouza@umassmed.edu (T.D.); Anand.Desai@umassmed.edu (A.D.); Silvia.Corvera@umassmed.edu (S.C.); 2 Division of Cardiovascular Medicine, Department of Medicine, University of Massachusetts Medical School, Worcester, MA 01605, USA; Heather.Learnard@umassmed.edu (H.L.); Shashi.Kant@umassmed.edu (S.K.); John.Keaney@umassmed.edu (J.F.K.J.); 3 Human Nutrition, Food, and Exercise Department, Virginia Tech, Blacksburg, VA 24060, USA

**Keywords:** adipose tissue, angiogenesis, exercise, glucose homeostasis, high-fat diet, metabolism

## Abstract

Exercise mitigates chronic diseases such as diabetes, cardiovascular diseases, and obesity; however, the molecular mechanisms governing protection from these diseases are not completely understood. Here we demonstrate that exercise rescues metabolically compromised high fat diet (HFD) fed mice, and reprograms subcutaneous white adipose tissue (scWAT). Using transcriptomic profiling, scWAT was analyzed for HFD gene expression changes that were rescued by exercise. Gene networks involved in vascularization were identified as prominent targets of exercise, which led us to investigate the vasculature architecture and endothelial phenotype. Vascular density in scWAT was found to be compromised in HFD, and exercise rescued this defect. Similarly, angiogenic capacity as measured by ex vivo capillary sprouting was significantly promoted with exercise. Together, these data demonstrate that exercise enhances scWAT vascularization and functional capacity for angiogenesis, and can prevent the detrimental effects of HFD. The improvement in these indices correlates with improvement of whole-body metabolism, suggesting that scWAT vascularization may be a potential therapeutic target for metabolic disease.

## 1. Introduction

Exercise is a powerful strategy to mitigate chronic diseases such as diabetes, cardiovascular diseases, and obesity. As these cardiometabolic diseases affect more than 25% of adults worldwide [1], finding molecular pathways that are important for disease prevention is paramount to improve outcomes and reduce the social and economic burden of disease. Exercise has proven to be an effective therapy for cardiometabolic diseases, however the tissue-specific molecular mechanisms important for disease prevention, particularly in conditions of Western eating habits (high fat diets), are still under investigation.

Recent studies have highlighted a major role for adipose tissue (AT) in either promoting or preventing cardiometabolic diseases. Adipose tissue is classically categorized as white adipose tissue (WAT) and brown adipose tissue (BAT) [2]. WAT depots contain large lipid droplets that function as storage repositories for triglycerides, whereas BAT is metabolically active, full of mitochondria, and high in expression of uncoupling protein 1 (Ucp1), which uncouples respiration from ATP production, resulting in heat production. There are multiple WAT depots, but the primary divisions are the visceral WAT (vWAT) surrounding the internal organs, and the subcutaneous WAT (scWAT) located beneath the skin [3]. Both the scWAT and the vWAT undergo negative changes with HFD [4] and adaptive metabolic changes with chronic exercise [5]. With chronic exercise, the scWAT expresses markers of BAT such as Ucp1; this reprogramming process is termed beiging or britening [6,7,8,9,10]. The functional significance of scWAT was highlighted as transplantation of scWAT, but not vWAT, from exercised mice to HFD mice improved glucose tolerance in the recipient mice [10].

Vascularization is important for the health of the adipose tissue and may be responsible, in part, for mediating global metabolic homeostasis. Due to the nature of WAT as a repository for lipids, it is unique in its ability to rapidly expand in response to increased nutrient intake. Tissue expansion requires healthy and functional vascularization to support the increased demands for oxygen, nutrition, and immune maintenance, suggesting that vascularization is important in defining WAT depot health and whole-body metabolism [11]. Importantly, the ability of scWAT to expand its vasculature is impaired with morbid obesity and this deficiency in vascularization largely correlates with insulin resistance [12]. Taken together, these data suggest that promoting scWAT angiogenesis may be a potential therapeutic strategy to mitigate obesity-related metabolic dysfunction. The purpose of the current study was to identify gene programs that are negatively impacted by HFD, but can be rescued by exercise, and then assess whether these gene programs are important for determining the scWAT phenotype.

## 2. Results

### 2.1. Exercise Improved Glucose Homeostasis in HFD Mice

**Figure 1:**
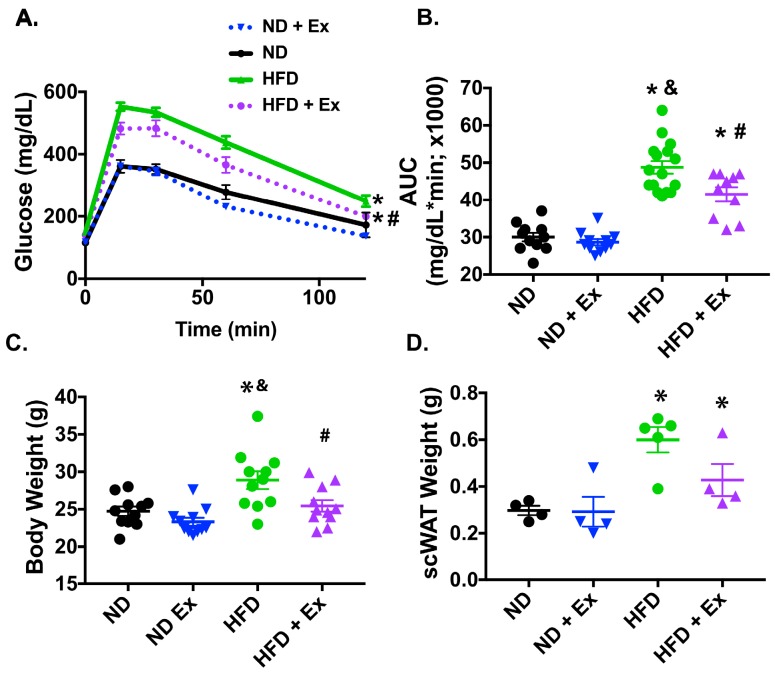
Exercise adaptation promotes glucose homeostasis (**A**): A glucose tolerance test (GTT) was performed over 120 min and (**B**): The GTT area under the curve (AUC) was calculated for each group of mice. (**C**) Body weight (g) was measured. (**D**): scWAT weight was measured. * *p* < 0.05 vs. ND; # *p* < 0.05 vs. HFD; & *p* < 0.05 vs. ND + Ex.

Global glucose homeostasis is known to be impaired with high fat diet feeding [13] and is significantly improved with exercise through multiple exercise adaptations [14]. Here we used a 45% HFD and a chronic exercise regimen (Supplementary Figure S1) to investigate the impact of diet and exercise on glucose homeostasis. The HFD impaired glucose handling which was significantly mitigated by exercise, as demonstrated by improved glucose tolerance (Figure 1A,B). In addition, the exercise mitigated the increased weight seen with the HFD and decreased the weight of the scWAT depot (Figure 1C,D).

### 2.2. Metabolic Gene Expression Was Improved with Exercise in HFD scWAT

**Figure 2:**
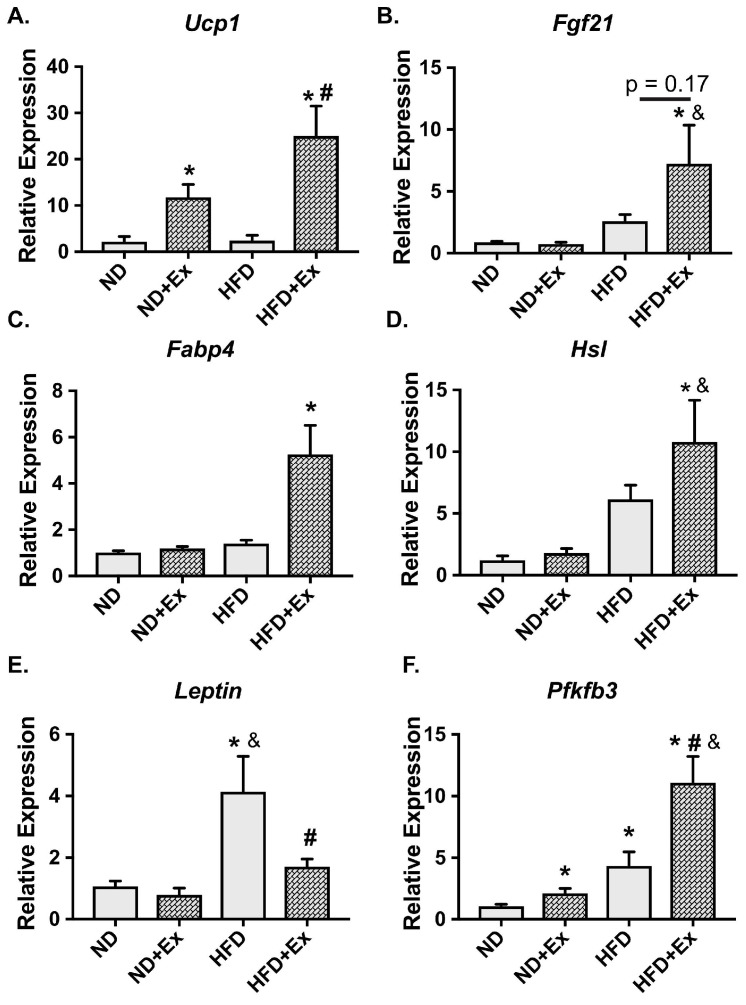
Metabolic gene expression in scWAT is improved with exercise. (**A**–**F**): scWAT was harvested and mRNA expression was measured using qPCR (* *p* < 0.05 vs. ND; # *p* < 0.05 vs. HFD; & *p* < 0.05 vs. ND + Ex; N = 5–7 mice/group).

We examined genes known to be important in exercise adaptation in scWAT. A gene involved in beiging/britening of WAT, *Ucp1*, was significantly augmented with exercise in the ND and HFD groups (Figure 2A). In addition, fibroblast growth factor 21 (*Fgf21*), was significantly increased in the HFD + Ex mice, but was not changed in the normal diet (ND) + Ex group (Figure 2B). We then examined genes that regulate scWAT metabolism. Genes involved in lipid metabolism and transport that are activated by *β*-adrenergic signaling in adipose tissue such as hormone sensitive lipase [15] (*Hsl*; Figure 2C) and fatty acid binding protein 4 [16] (*Fabp4;* Figure 2D) increased with exercise in the HFD mice. HFD significantly increased leptin expression which was corrected by exercise (*Lep*; Figure 2E). Finally, expression of 6-phosphofructo-2-kinase/fructose-2,6-biphosphatase 3 (*Pfkfb3*), a gene shown to be protective from diet-induced insulin resistance when overexpressed in adipose tissue [17], was significantly increased in the HFD mice with exercise (Figure 2F). Taken together, these results demonstrate that the chronic exercise regimen results in adaptive beiging and metabolic responses in scWAT. However, these measures did not pinpoint pathways that were negatively influenced by diet and repaired by exercise.

### 2.3. scWAT Gene Programs Involved in Vascularization Were Rescued by Exercise

**Figure 3:**
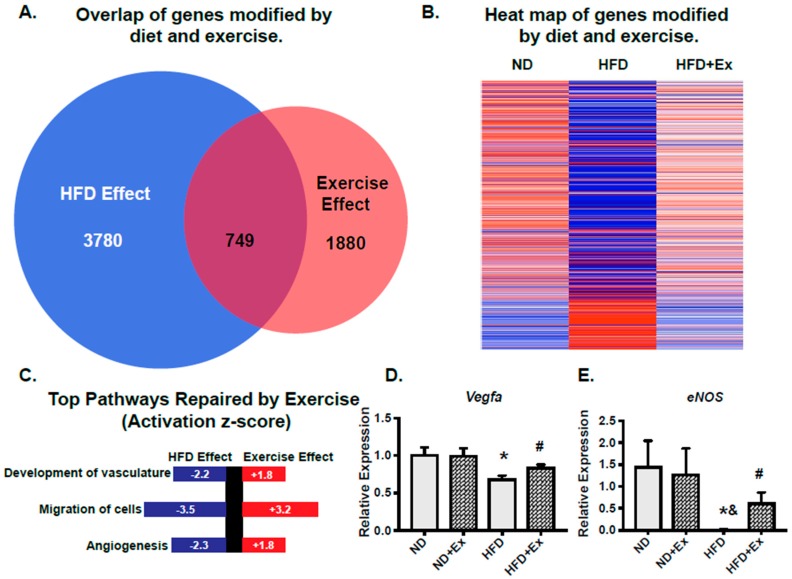
Exercise rescues HFD scWAT transcriptomic pathways involved in angiogenesis. (**A**): Transcripts significantly altered by diet (HFD/ND; *p* < 0.05) were compared to transcripts significantly altered with exercise (HFD + Ex/HFD; *p* < 0.05). (**B**): Heat map of the genes modified by both diet and exercise. (**C**): Gene pathways were examined by Ingenuity Pathway Analysis (IPA). From this analysis, the top 3 pathways suppressed with HFD and recovered by exercise were determined. P-value and activation z-score which predicts whether a specific disease or biological function is increased (positive z-score) or decreased (negative z-score) based on the experimental dataset are shown. (**D**,**E**): mRNA expression from scWAT was measured by qPCR (* *p* < 0.05 vs. ND; # *p* < 0.05 vs. HFD; & *p* < 0.05 vs. ND + Ex; N = 5–7 mice/group).

To interrogate which gene programs were negatively influenced by diet and could be reversed by exercise, we used transcriptional profiling. Specifically, we identified transcripts that were significantly modified by diet (HFD effect; *p* < 0.05 HFD compared to ND) and then overlaid these with transcripts that were significantly modified by exercise in HFD (Exercise Effect; *p* < 0.05 HFD Ex compared to HFD; Figure 3A). The HFD effect uncovered 3780 genes that were significantly changed versus the ND group (*p* < 0.05). Of these, 2036 genes were suppressed and 1744 were increased in expression. Exercise significantly (*p* < 0.05) modified 1880 genes. There were 750 genes significantly decreased and 1130 significantly increased by exercise in the HFD group. Examining the transcripts that were modified by *both* diet and exercise uncovered 749 transcripts as shown in the heat map (Figure 3B), with red indicating increased expression and blue indicating decreased expression. Differentially expressed genes were further analyzed using Ingenuity Pathway Analysis (IPA) to define the most enriched biological functions. IPA makes predictions about affected cellular processes and biological functions (Diseases and Functions) and the directional change on that effect. The top 3 *Disease and Functions* identified were “Development of Vasculature” (*p* = 7.6 × 10^−16^), “Migration of Cells” (*p* = 9.2 × 10^−6^), and “Angiogenesis” (*p* = 9.2 × 10^−16^) (Figure 3C). Supplementary Table S1 shows the top ten enriched terms corresponding to Diseases and Function along with the overlapping number of genes in the experimental dataset, *p*-value and activation z-score. We also examined the growth factor upstream regulators (Supplementary Table S2). Through transcriptomic pathway profiling, these data show that vascularization of the scWAT is a major target of exercise in mice fed an HFD.

Based on these findings, we examined two prominent genes involved in vascular health and angiogenesis: vascular endothelial growth factor a (*Vegfa*) and endothelial nitric oxide synthase (*Enos*). *Vegfa* is important in initiating angiogenesis in scWAT and mediating beneficial effects of glucose homeostasis [18]. In addition, our pathway analysis determined that the *Vegfa* pathway was an important upstream regulator (Supplementary Table S2) of the HFD rescue by exercise. Our results demonstrate that *Vegfa* expression was significantly suppressed by HFD and rescued by exercise (Figure 3D). *Enos* is also important in vascular health and angiogenesis, and has been shown to be important in exercise-induced adaptation in the scWAT [9]. We found that HFD suppressed *Enos* expression while exercise rescues this suppression (Figure 3E). Together, these data indicate that an HFD impairs pathways involved in scWAT vascular health and that a chronic exercise regimen rescues these gene expression changes.

### 2.4. Vascularization and Functional Angiogenesis are Enhanced in scWAT with Exercise

**Figure 4:**
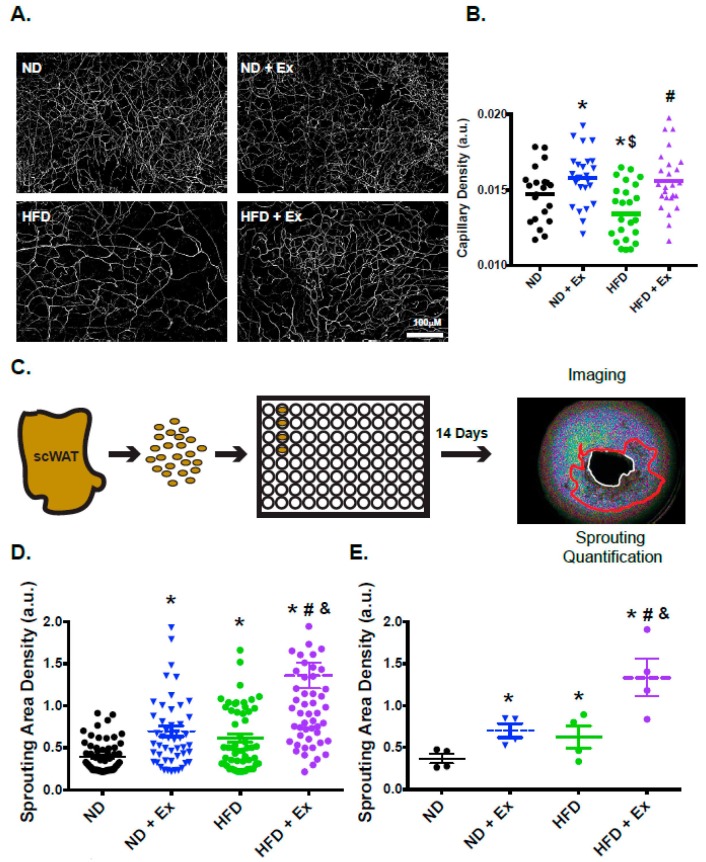
Exercise improves vascular density and increases angiogenic capacity in scWAT. (**A**): Maximal intensity projections of 25 epiflourescent images acquired at 10-μm intervals (250-μm thickness) of isolectin B4 stained fragments of scWAT from mice fed ND (top left panel), ND + Ex (top right panel) HFD (bottom left panel) or HFD + Ex (Bottom right panel). (**B**): Data from the micrographs was quantified demonstrating vascular density in the scWAT depots. (5 sections/mouse; *N* = 5 mice/group). (**C**): An angiogenesis sprouting assay was used to measure sprouting (angiogenic) capacity. scWAT depots were carefully dissected and cut into 1mm segments. These segments were then plated in a 96-well matrigel coated plates and after 14 days of growth, imaged and sprouting was quantified. A representative image of explant out-growth (circled in red) is shown on the right. (**D**): Angiogenic capacity ((number of sprouts/explant)(% of explants sprouting)) of explants derived from mice fed ND, ND + Ex, HFD, HFD + Ex. Angiogenesis was quantified as area of outgrowth. (**E**): Summary data is shown for each mouse. (>25 segments/mouse were assessed and 4–5 mice/group measured (* *p* < 0.05 vs. ND; # *p* < 0.05 vs. HFD; & *p* < 0.05 vs. ND + Ex).

Our transcriptomic profiling indicated that expression of genes associated with vascular growth was impaired with HFD, consistent with previous studies that documented impaired vascularization of WAT with HFD [19]. To determine if exercise was able to modify the scWAT vascular phenotype we used endothelial staining (isolectin B4) to investigate vessel density of the WAT depots. Compared with the ND mice, the HFD mice had significantly less scWAT vessel density (Figure 4A). However, there was a significant increase in scWAT vessel density and improved architecture in the HFD exercised mice compared with the HFD mice (Figure 4A). Quantification of vascular density demonstrated a significant decrease in vessel density with HFD; the exercise regimen returned vascular density to a level similar to ND samples (Figure 4B). Interestingly, the ND + Ex mice also demonstrated increased capillarization. Furthermore, improved vascular density was also observed in visceral WAT (vWAT; Supplementary Figure S2). These data demonstrate that vascular density was increased with exercise in both ND and HFD mice.

To determine if functional vascularization was modified by exercise, the angiogenic potential of the scWAT was assessed. To address this question, we used an ex vivo explant assay previously developed by our group [20] (Figure 4C). Ex vivo measurement of angiogenesis requires small explants to be plated in matrigel for 2 weeks. Over time we are then able to observe endothelial outgrowth [20]. With the diet alone there was no significant difference. However, there was a significant increase in explant outgrowth observed with exercise (Figure 4D,E). Individual explants were quantified (Figure 4D) and summarized per group (Figure 4E), demonstrating a significant increase in angiogenic potential due to exercise. Therefore, not only was total vascularization improved in HFD exercised mice, but the ex vivo angiogenic potential of the scWAT depots was also enhanced by exercise.

## 3. Discussion

Exercise provides protection from diet-induced metabolic defects as demonstrated by improved glucose homeostasis and metabolic reprogramming of the scWAT. In HFD mice, exercise specifically rescued gene programs involved in vascular health and angiogenesis. Examining the scWAT phenotype, we found significant improvements in vascular integrity, density, and angiogenic potential in the HFD exercised mice. Intriguingly, exercise improved vascular density and the angiogenic potential of scWAT in both ND and HFD, suggesting that the effect of exercise on scWAT vascularization may be an important adaptive response. In summary, we demonstrate that transcriptomic networks important in vascular expansion are negatively influenced by HFD and can be effectively reversed by chronic endurance exercise. The increased vascularization and enhanced angiogenic potential we observed with exercise are likely involved in mediating metabolism and glucose homeostasis.

The focus of the current study was to identify pathways that were rescued in scWAT with exercise, and to determine whether these pathways were important in mediating scWAT phenotype. Importantly, we found that the most prominent biologic functions that were reversed with exercise in HFD were angiogenic pathways; these were functionally significant as we observed improved angiogenic capacity in the scWAT. Furthermore, we observed that there was no difference in the angiogenic capacity of the vWAT, suggesting that scWAT may play a more prominent role in the adaptive response to exercise. A recent study documented increased vascularization in rat WAT with exercise, further indicating the adaptive role of scWAT [21]. This notion is consistent with the study by Stanford et al. [10] which demonstrated that scWAT was specifically important in the beneficial responses to exercise as transplantation of exercised scWAT, but not vWAT, to non-exercised controls improved glucose homeostasis. Therefore, the beneficial effects of exercise may, in part, be linked through the functional angiogenic capacity of the scWAT.

Our study supports the idea that adipose tissue vascular health and angiogenesis may be important therapeutic targets of exercise. Vascularization is important in dictating AT and whole-body health as demonstrated by devascularization (rarefaction) of BAT. BAT is normally highly vascularized and is involved in regulating glucose homeostasis. However, its rarefaction resulted in whitening and impaired glucose tolerance [22]. Conversely, increased vascularization through overexpression of VEGF-A in WAT depots improved whole-body metabolism [23,24]. In fact, localized tissue delivery of VEGF-A to scWAT promoted angiogenesis, induced beiging/britening and enhanced survival after transplantation [18]. Furthermore, transient overexpression of VEGF-A in adipose tissue was recently shown to activate the sympathetic nervous system to promote energy expenditure [25]. Our results demonstrate that *Vegfa* expression and vascular density were significantly suppressed by HFD and rescued by exercise, suggesting that the increased vascularization may contribute to the improved glucose homeostasis observed in our study. In addition, upstream regulators identified by pathway analysis highlighted vascular growth factors (Supplementary Table S2). Together, these data suggest that the amount, integrity, and angiogenic potential of adipose tissue vascularization are important in mediating local and global metabolism and this may be a key aspect of the adaptive benefits of exercise.

Numerous studies have documented vascular changes related to eNOS in various tissues with exercise. For example, consistent exercise improves vascular function in large conduit vessels such as the aorta, largely through increased expression and activity of eNOS [26]. Increased eNOS may also be responsible for mediating decreases in blood pressure seen with chronic exercise [27]. *Enos* is necessary for improved WAT angiogenesis [28], and global overexpression of *Enos* improved diet-induced glucose intolerance [29]. Moreover, exercise-induced mitochondrial biogenesis and glucose uptake in the scWAT require *Enos*, as *Enos* knockout mice are unresponsive to these beneficial effects of exercise [9]. We found that HFD suppressed *Enos* expression while exercise rescues this suppression, suggesting that chronic exercise maintains *Enos* expression, which may lead to the improved vascular integrity and enhanced angiogenic potential documented in our study.

The data presented here focused on scWAT angiogenesis. However, changes in other organs have noted global endothelial responses. Improved vascularization has been associated with increases in skeletal muscle collateralization [30] and angiogenesis [31]. Indeed, chronic exercise increases circulating endothelial progenitor cells which was demonstrated to promote vascular repair after vascular injury [32]. Together these data suggest that exercise may improve vascular health by initiating global endothelial responses. Our present study has also demonstrated that there are tissue-specific endothelial changes; exercise changed the inherent phenotype of endothelial cells in scWAT, as shown by the increased sprouting that occurred *ex vivo*. Taken together, these data indicate that tissue specific endothelial populations may be important for the salutary benefits of exercise. Future studies will be essential to elucidate the relationship between global and local responses of different vascular beds to exercise, and to determine the relative importance of the WAT vascular phenotype in protection from disease.

## 4. Materials and Methods

### 4.1. Animals and Diets

Male C57Bl/6J mice (Jackson Laboratories, Bar Harbor, ME, USA) were used for all studies. The mice (10–12 weeks of age) were randomized to either a standard normal diet (ND) containing 14.3% of calories from fat or a high-fat diet (HFD) containing 45% of calories from fat (TD.06415; Harlan Teklad). The metabolizable energy from the ND was 3.14 kcal/gm and 4.6 kcal/gm for the HFD. The mice were then randomly assigned to exercise (ND + Ex or HFD + Ex) for 7 weeks. All groups were allowed to eat ad libitum throughout the duration of the study (except during the time periods of exercise/sedentary). The animals were housed on a 12:12-h light-dark cycle in a temperature-controlled room at 25 °C. The University of Massachusetts Medical School Institutional Animal Care and Use Committee approved all procedures (A-3306-01, 27 November 2017).

### 4.2. Exercise Intervention

Exercise was conducted on a motorized treadmill (Columbus Instruments Model #1055-SRM-D58, Columbus, Ohio, Columbus, OH, USA). All mice were acclimated to the treadmill 3 days before exercise regimens. On Day 1, the mice were allowed to stand on the treadmill for 15 min. On Day 2, the mice walked on the treadmill at 5 m/min for 15 min. On Day 3, the mice began walking at 5 m/min and the treadmill speed was gradually increased to 15 m/min and the mice ran at this speed for 15 min.

The chronic exercise training consisted of treadmill running for 30 min/day at 18 m/min (~70% VO_2_ max) [33] 5 days/week for 7 weeks. This protocol was modified from similar protocols that have shown skeletal muscle and vascular adaptation to exercise [34,35,36]. We confirmed this exercise protocol using an exercise to exhaustion test (see below) which demonstrated improved running distance to exhaustion in the exercised groups (Supplementary Figure S1). To control for any non-exercise effects of treadmill running (handling, novel environment, noise, and vibration), the non-exercised group of mice (sham exercise) were placed on the top of the treadmill apparatus for an identical period of time. The mice were not subjected to electric shock during the treadmill sessions to avoid stress. Tissue was harvested >24 h following the last exercise bout.

The exhaustive exercise began at 5 m/min for 15 min followed by gradual increases in speed at 3 min intervals until the treadmill speed reached 24.25 m/min, at which point it was held at this speed for 30 min or until the mice reached exhaustion. The state of exhaustion was established by a mouse remaining in the lower ¼ of the treadmill 3 cumulative times despite gentle encouragement.

### 4.3. Glucose Tolerance Test

A glucose tolerance was test was performed on mice at baseline and after 6 weeks of exercise (>24 h after last bout of exercise). The mice were then fasted for 12 h and glucose (2 g/kg) was delivered by intraperitoneal injection. Blood samples were harvested from the tail vein at the indicated times, and glycaemia was determined using a Bayer Breeze 2 glucometer. Data were plotted as milligrams (mg)/deciliters (dl) over time and area under the curve (AUC).

### 4.4. Ex Vivo Angiogenesis Assay of Adipose Tissue

Explants from freshly harvested mouse scWAT were prepared and embedded as described [20]. In brief, after removal of obvious vasculature, the tissue was cut into ~1-mm^3^ pieces, which were then embedded in individual wells of standard 96-well cell culture plate containing 40 μL of growth factor–depleted Matrigel (Corning, Corning, NY, USA). The Matrigel was allowed to polymerize at 37 °C for 30 min, after which wells were filled with 200 μL of EBM-2 media supplemented with microvascular endothelial growth factors (EGM-2 MV) (Lonza, Basel, CH), and one-half of the media were replaced every second day. More than 25 explant pieces from each mouse were embedded for each treatment condition for the analysis. Phase contrast images were acquired using a Zeiss Axio Observer Z1 equipped with an automated stage and a Clara High Resolution CCD Camera (Andor, Concord, MA, USA). Images were taken under 2.5× magnification as a stack composed of five Z planes at 150-μm intervals and as a canvas of four quadrants per well, and then combined into a single three-dimensional image. Subsequent image processing and quantification of capillary growth were determined as described in Rojas-Rodriguez et al. [20]. Image processing for quantification of angiogenic capillary sprouting included background subtraction, average intensity projection of the Z-stack and binarization using the ImageJ open platform.

### 4.5. Whole Mount Tissue Staining

Fragments of scWAT were fixed in 4% formaldehyde and extensively washed in 1X PBS, permeabilized with Triton X-100 and then incubated for 1 h in the presence of Alexa 488-conjugated IB4 (1:50, Invitrogen (Life Technologies, Carlsbad, CA, USA). Negative controls treated with irrelevant mouse IgG and then processed in parallel with stained sections. All tissue was counterstained with Hoechst 33342 (Life Technologies, Grand Island, NY, USA). Images of vascular architecture were assessed by two blinded examiners and classified into groups. Image J was used for total vascular quantification.

### 4.6. Quantitative Real-Time PCR

Tissue was harvested and total RNA was extracted using TRIzol (Invitrogen (Life Technologies), Carlsbad, CA, USA). For quantitative mRNA analysis, 1–2 μg of the total RNA was reverse-transcribed using an iScript cDNA synthesis kit (Bio-Rad, Hercules, CA, USA). Quantitative real-time PCR (RT-PCR) analysis was performed using an iQ SYBR Green supermix kit or TaqMan probes and RT-PCR detection system following the manufacturer’s instructions (MyiQ, Bio-Rad and TaqMan, Life Technologies, Carlsbad, CA, USA). Ferritin heavy chain, β-actin, and hprt were used as internal housekeeping genes. Relative gene expression was calculated by the 2^−ΔΔCt^ method.

### 4.7. Microarray Analysis

scWAT was harvested from age-matched male mice (17–19 weeks), 3 biological replicates were used in each group (ND, ND + Ex, HFD, HFD + Ex). RNA from scWAT concentrations were determined using a Nanodrop 2000 Spectrophotometer (Thermo Fisher, Wilmington, DE, USA). The RNA quality was assessed using an Agilent 2100 Bioanalyzer (Agilent Technologies, Santa Clara, CA, USA). Only samples with an RNA integrity number above 7.5 and normal 18-s and 28-s fractions on microfluidic electrophoresis were used. Second- strand cDNA was then labeled with the Affymetrix WT terminal labeling kit, and samples were hybridized to Affymetrix Mouse Gene 1.0 ST arrays (Affymetrix, Santa Clara, CA, USA). Microarray data was analyzed using Affymetrix Transcriptome Analysis Console (TAC) Software (ThermoFisher; https://tools.thermofisher.com/content/sfs/brochures/tac_software_datasheet.pdf) which integrates the established linear modeling for microarrays (LIMMA; [37]) package, using R/Bioconductor software to provide an integrated solution for analyzing data from gene expression experiments, to calculate fold change and significance. Pathway analysis of the transcriptomic data was conducted using Ingenuity Pathway Analysis (IPA; Qiagen, Hilden, DE, USA).

### 4.8. Statistical Analysis

Results are expressed as means ± SEM. Data were analyzed by GraphPad Prism 7.0 software (GraphPad Software, La Jolla, CA, USA) using unpaired Student *t* test, one-way analysis of variance (ANOVA), or two-way ANOVA with Newman-Keuls post hoc test, as appropriate.

## Supplementary Materials

Supplementary materials can be found at https://www.mdpi.com/1422-0067/20/8/2046/s1.
