# Supplementary material for: Exercise Rescues Gene Pathways Involved in Vascular Expansion and Promotes Functional Angiogenesis in Subcutaneous White Adipose Tissue
Source: Int J Mol Sci. 2019 Apr 25;20(8):2046. doi: 10.3390/ijms20082046 (PMC6515240; doi:10.3390/ijms20082046)
Supplement: Supplementary file 1 [file ijms-20-02046-s001.pdf]

Supplementary Figure 1: Exercise to Exhaustion: Mice were exercised to exhaustion and total distance run in meters (M) was recorded (* $p < 0.05$ vs ND; # $p < 0.05$ vs HFD; & $p < 0.05$ vs ND + Ex).

Supplementary Figure 1

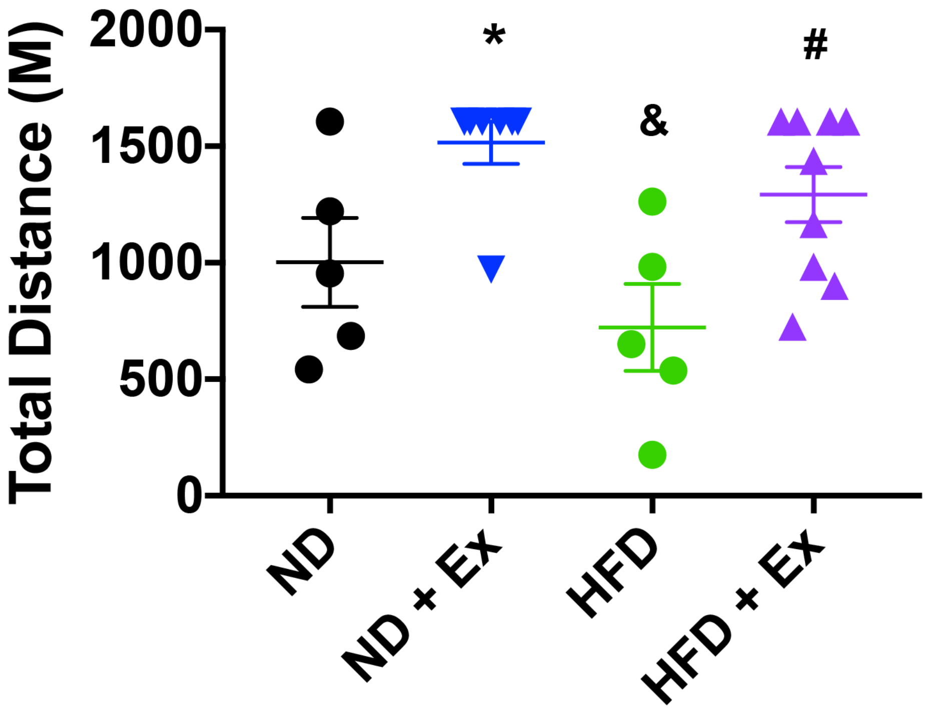

Supplementary Figure 2: Exercise improves vascular density but not angiogenic capacity in vWAT. **A:** Maximal intensity projections of 25 epiflourescent images were acquired at 10 μm intervals of isolectin B4 stained sections of scWAT from mice fed ND (top left panel), ND + Ex (top right panel) HFD (bottom left panel) or HFD + Ex (bottom right panel) **B:** Data from the micrographs were quantified to demonstrate vascular density in the scWAT depots. (5 sections/mouse; N = 5 mice/group). **C:** Angiogenic capacity was measured as in Figure 4. Angiogenesis was quantified as area of outgrowth. **D:** Summary data is shown for each mouse. (>25 segments/mouse were assessed (*p < 0.05 vs ND; # p < 0.05 vs HFD; & p < 0.05 vs ND + Ex).

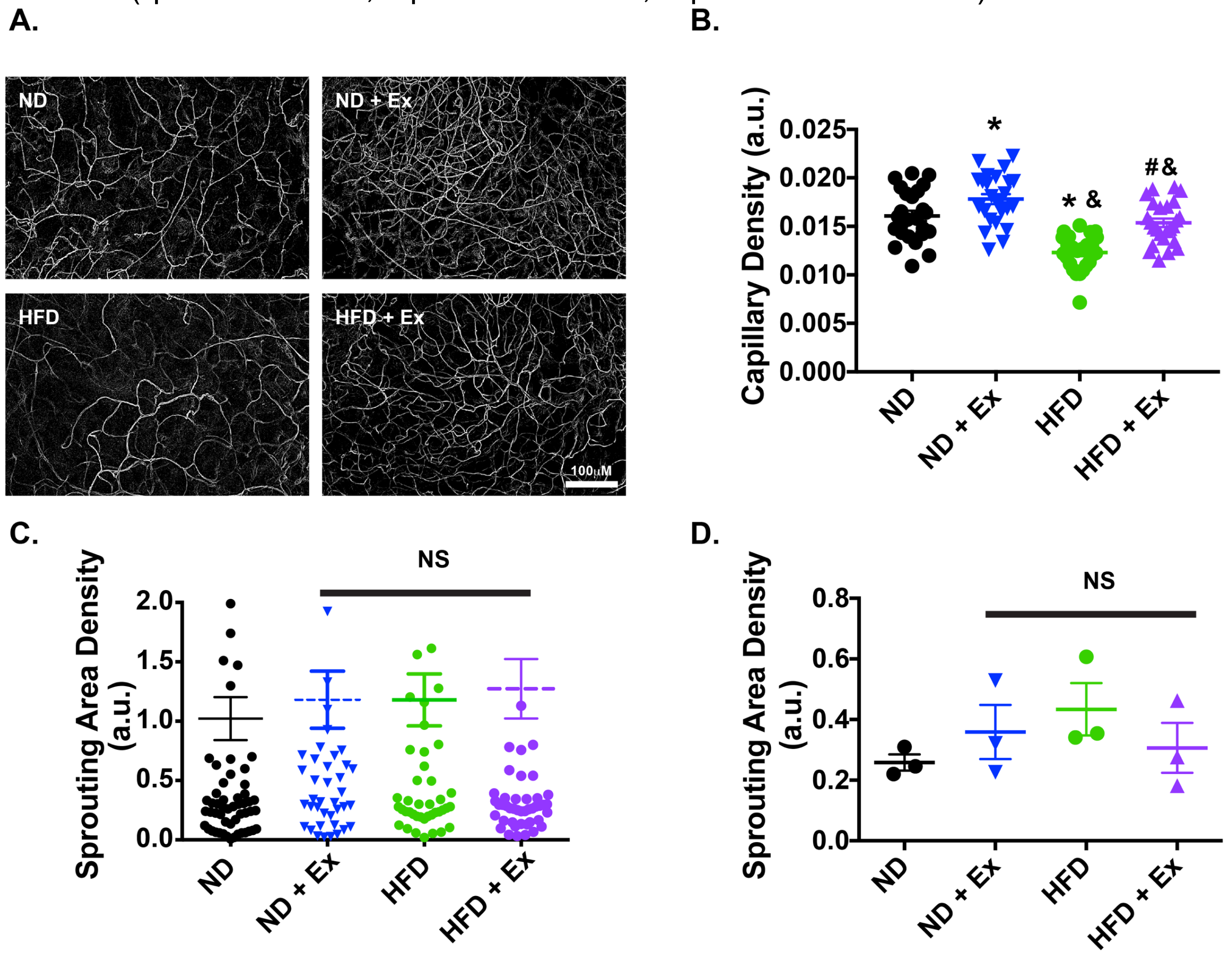

Supplementary Figure 2

Supplementary Table 1: The top 10 Ingenuity Pathway Analysis (IPA) defined most enriched biological functions (Disease and Biological Functions) and the directional change of that effect. Pathways negatively influenced by diet and reversed by exercise have been highlighted in red.

**IPA Diseases and Biological Functions**			**HFD to ND**	**HFD + Ex to HFD**
Categories:	*Annotation:*	*p-value:*	*Activation z-score:*	*Activation z-score:*
Cardiovascular System Development and Function	**Development of vasculature**	7.61E-16	**-2.254**	**1.835**
Cellular Movement	**Migration of cells**	9.2E-16	**-3.462**	**3.164**
Cardiovascular System Development and Function,Organismal Development	**Angiogenesis**	9.27E-16	**-2.256**	**1.837**
Organismal Injury and Abnormalities	Benign lesion	3.07E-15	0.518	-0.876
Cell Death and Survival	Necrosis	3.06E-14	-0.029	-0.563
Cellular Movement	**Cell movement**	3.59E-14	-3.194	3.13
Cell Death and Survival	Cell death	6.79E-14	0.615	-0.843
Metabolic Disease	Glucose metabolism disorder	8.02E-14	-0.062	-1.18
Cancer,Organismal Injury and Abnormalities	Benign neoplasia	1.11E-13	0.926	-0.926
Cardiovascular System Development and Function,Organismal Development	**Vasculogenesis**	2.6E-13	**-2.481**	**2.764**

Supplementary Table 2: IPA defined upstream growth factors.

IPA Upstream Regulator		HFD to ND	HFD + Ex to HFD
Growth Factors:	*p-value of overlap:*	*Activation z-score:*	*Activation z-score:*
EGF	0.000000774	-3.31	3.068
IGF1	0.00000322	-2.112	2.5
FGF2	3.39E-08	-1.794	2.152
TGFA	0.00181	-2.025	2.025
PDGFC	0.0024	-1.948	1.948
CTGF	0.000677	-2.586	1.943
TGFB1	7.17E-19	-1.974	1.873
NGF	0.035	-2.675	1.836
IGF2	0.00259	-1.809	1.809
FGF7	0.0121	-1.633	1.633
AGT	0.000773	-2.221	1.596
BMP2	0.00151	-1.497	1.497
HGF	0.0108	-1.932	1.406
GRP	0.00466	-1.257	1.257
NRG1	0.00141	-1.237	1.237
KITLG	0.0122	-1.211	1.211
VEGFA	0.0159	-2.184	1.194
NOG	0.0132	1.253	-1.253
WISP2	0.000064	1.551	-1.551
